# New Endemic *Legionella pneumophila* Serogroup I Clones, Ontario, Canada

**DOI:** 10.3201/eid1603.081689

**Published:** 2010-03

**Authors:** Nathalie Tijet, Patrick Tang, Mya Romilowych, Carla Duncan, Victoria Ng, David N. Fisman, Frances Jamieson, Donald E. Low, Cyril Guyard

**Affiliations:** Ontario Agency for Health Protection and Promotion, Toronto, Ontario, Canada (N. Tijet, P. Tang, M. Romilowych, C. Duncan, D.N. Fisman, F. Jamieson, D.E. Low, C. Guyard); Research Institute of the Hospital for Sick Children, Toronto (V. Ng, D.N. Fisman); Australian National University, Canberra, Australian Capital Territory, Australia (V. Ng); University of Toronto, Toronto (F. Jamieson, D.E. Low, C. Guyard); Mount Sinai Hospital, Toronto (F. Jamieson, D.E. Low, C. Guyard)

**Keywords:** Legionella pneumophila serogroup 1, molecular epidemiology, sequence-based typing, phylogeny, clonal analysis, emerging clones, bacteria, Canada, research

## Abstract

The water-borne pathogen *Legionella pneumophila* serogroup 1 (Lp1) is the most commonly reported etiologic agent of legionellosis. To examine the genetic diversity, the long-term epidemiology, and the molecular evolution of Lp1 clinical isolates, we conducted sequence-based typing on a collection of clinical isolates representing 3 decades of culture-confirmed legionellosis in Ontario, Canada. Analysis showed that the population of Lp1 in Ontario is highly diverse and combines lineages identified worldwide with local strains. Identical types were identified in sporadic and outbreak-associated strains. In the past 15 years, the incidence of some lineages distributed worldwide has tended to decrease, and local endemic clones and lineages have emerged. Comparative geographic distribution analysis suggests that some lineages are specific to eastern North America. These findings have general clinical implications for the study of Lp1 molecular evolution and for the identification of Lp1 circulating strains in North America.

## Abstract 2

Identifying geographic distribution can improve surveillance and clinical testing procedures.

*Legionella* species are implicated in 2 clinical syndromes: Legionnaires’ disease (LD) and Pontiac fever, which are collectively known as legionellosis. Pontiac fever is a self-limited, influenza-like illness, whereas Legionnaires’ disease is a common cause of serious bacterial pneumonia (*1*,*2*).

Among the 52 species and 70 serogroups of *Legionella* species (*3*), *L. pneumophila* is the major cause of sporadic and outbreak legionellosis (91.5%), and serogroup 1 is the predominant serotype (84.2%) (*4*). In industrialized countries, *L. pneumophila* is the second most common pathogen detected in cases of community-acquired pneumonia that requires patient admission to intensive care units (*5*,*6*).

During an outbreak or after the detection of sporadic cases, appropriate identification and typing methods are essential for epidemiologic investigations. Adequate typing methods are also crucial to determine the degree of relatedness of bacteria and to enable the reconstruction of microevolutionary events (*7*). On the basis of analysis of 7 loci, a standard sequence-based method for the typing of *L. pneumophila* serogroup 1 (Lp1) was developed by the European Working Group for *Legionella* Infections (EWGLI) (*8*,*9*).

In previous population-based studies, a *Legionella* sequence-based typing (SBT) scheme was used to analyze clinical strains either from Europe or with limited time-span coverage (*10*–*12*). In the present study, we applied SBT to examine the genetic diversity, the long-term epidemiology, and the molecular evolution of Lp1 clinical isolates using a population-based collection that encompassed isolates from 30 years of culture-confirmed legionellosis cases in Ontario.

## Methods

### Source of Isolates

Legionellosis is a notifiable disease in Ontario (population 13 million persons). Since 1978, the diagnosis of *Legionella* infections has been centralized at the Ontario Public Health Laboratory (OPHL). This laboratory serves as the *Legionella* reference laboratory and performs all testing for outbreak investigations and most testing of clinical specimens. Therefore, isolates analyzed in this study are representative of the strains isolated in Ontario in the past 3 decades. Information available in the Ontario database includes dates of onset of illness, patient’s age and sex, and city and hospital or healthcare facility from which specimens were submitted (*13*). No specimens were submitted for *Legionella* isolation from 1978 through 1979. From 1980 through 1985, a mean ± SD of 424.1 ± 281.3 specimens was submitted for isolation every year. From 1986 through 2007, a mean ± SD of 1,783.5 ± 258.4 specimens was submitted for isolation every year. The mean ± SD number of Lp1 culture-confirmed cases/year during the study period was 7.4 ± 3.5. The proportion of culture-confirmed case-patients with *L. pneumophila* infection remained stable during the period of analysis, and 66% of the isolates were Lp1 (*13*).

Lung tissues, bronchial-alveolar lavage specimens, or sputum specimens were homogenized by using a tissue grinder, streaked on buffered charcoal yeast extract agar plates and incubated at 37°C (3–7 days). Species and serogroups were confirmed by direct immunofluorescent antibody assay and slide-agglutination (*14*,*15*). Isolates (n = 217) were stored at –80°C in trypticase soy broth supplemented with 5% horse blood. Twenty-three isolates obtained in 1996 and 1997 could not be used and were omitted from our analysis. Outbreaks were defined as >2 cases that were submitted from the same hospital or healthcare facility or with links to a common source with onset during the same 30-day period.

### Sequence-based Typing

SBT using loci *fla*A, *pil*E, *asd*, *mip*, *mompS*, *proA,* and *neuA* was conducted according to the EWGLI scheme (*8*,*9*). Automated contig-assembly and base-calling of DNA sequence traces were performed by using the EWGLI sequence quality tool (*16*). The sequences obtained from this work are available in the EWGLI-SBT database (www.ewgli.org)

### Phylogenetic and Allelic Diversity Analyses

Multiple sequence alignments of concatenated DNA sequences and phylogram construction were carried out with ClustalW2 (www.ebi.ac.uk/Tools/clustalw2/index.html) by using the neighbor-joining method with 1,000 bootstrap replicates (*17*). Clonal analyses were performed by using eBURST3 (http://eBURST.mlst.net) with a group definition set to 6 identical alleles and sequence type (ST) allelic profiles were clustered with the unweighted pair group method with arithmetic mean (UPGMA) algorithm by using splits Tree4 (*18*). The standardized index of association (*I_A_^S^*) and the mean genetic diversity were calculated with LIAN 3.5 (*19*).

### Ontario Health Regions and Rates Calculations

The 36 public health units of Ontario were aggregated into 7 health regions (OHRs) with populations ranging from ≈0.5 to 2 million persons: Toronto, South West, Central South, Central West, Central East, East, and North. Average rates were calculated by dividing disease counts by the Statistics Canada population estimates (*20*,*21*). OHR population estimates were not available before 1995 and were estimated from 1990 through 1995 and 2006 through 2007 by linear extrapolation.

### Statistical Analyses and Mapping

Statistical analyses were performed by using STATA (StataCorp, College Station, TX, USA). Thematic maps were created by using ArcGIS (ESRI, Redlands, CA, USA).

## Results

### Lp1 Sequence-based Typing

The 194 isolates, collected from 1978 through 2007, were resolved into 62 STs (Table A1). Seven STs were represented by at least 10 isolates, 13 STs consisted of groups containing 2–4 isolates, and 42 STs were represented by 1 isolate. In comparison to the EWGLI dataset, 42 STs have only been reported in North America, and 41 STs are unique to the province of Ontario. The ST with the largest number of isolates was ST1 (n = 31). STs previously reported in the EWGLI database and responsible for >9 cases in Ontario include ST36 (n = 10), ST37 (n = 21), ST42 (n = 10), and ST62 (n = 16). Two STs, specific to the province of Ontario, were detected in >9 legionellosis cases: ST211 (n = 15) and ST222 (n = 13). STs resolved from outbreak isolates were confirmed to be epidemiologically concordant since related isolates were assigned identical STs. ST211 strains were obtained from patients in 1 outbreak (n = 2) in 1993 and from 13 patients with sporadic cases. Seven of the 13 ST222 isolates were recovered from a legionellosis outbreak at a long-term care facility in Ontario in 2005 (*10*). ST226 was also differentiated from strains responsible for a suspected outbreak (n = 2) and is specific to Ontario.

Across the 7 loci, 99 alleles were identified. Three new alleles were found, 2 of which (*asd* 32 and *proA* 33) were identified in a ST357 strain isolated in 2002 from a patient with a sporadic case. The third new allele (*mompS* 52) was identified in a ST358 strain isolated in the South West OHR. At the individual loci level, the total number of alleles ranged from 10 at *flaA* to 21 at *mompS*. Because the population of Lp1 clinical isolates found in Ontario appeared to be distinct from the isolates reported in the EWGLI database, we performed linkage analyses and looked at genetic diversity of our dataset compared to that of the EWGLI dataset. Linkage analyses showed that the I_A_^S^ for the complete dataset was 0.4913. This value is comparable with the I_A_^S^ of 0.494 in previous studies and suggests linkage disequilibrium in the dataset obtained from the population of clinical Lp1 isolates in Ontario (*22*). With a value of 0.8041 ± 0.0155, the mean ± SD genetic diversity of our dataset was similar to the mean genetic diversity of the EWGLI dataset.

### Phylogenetic Analysis

**Figure 1:**
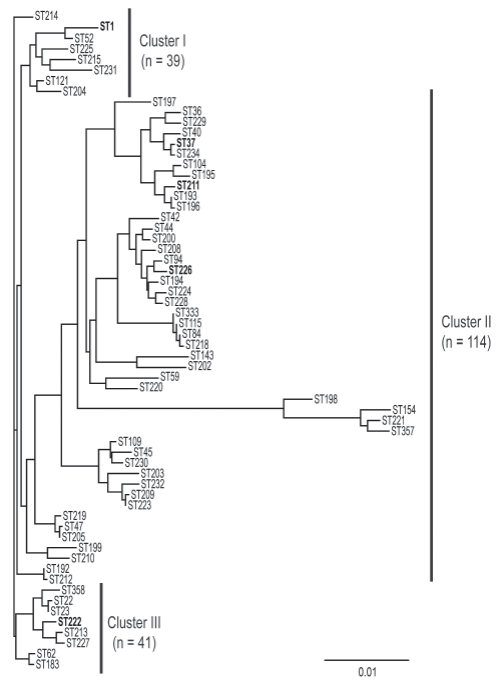
Phylogenetic analysis of *fla*A, *pi*lE, *asd*, *mip*, *mompS*, *proA,* and *neuA* concatenated sequences from the 62 *Legionella pneumophila* serogroup 1 sequence types (STs) identified in Ontario. The tree was constructed with ClustalW2 (www.ebi.ac.uk/Tools/clustalw2/index.html) and the neighbor-joining method with 1,000 bootstrap replicates. Scale bar indicates genetic distances between sequences. STs in **boldface** were detected in outbreaks.

The population structure of Lp1 clinical isolates from Ontario was analyzed by using 62 concatenated sequences of 7 loci and compared with results of cluster analysis deduced from SBT allelic profiles. Three major clusters were visually identified from the phylogenetic tree (Figure 1). All clusters contain isolates from outbreak and sporadic cases. None of the identified clusters or subgroups were exclusively formed with strains identified with STs specifically reported in Ontario. This suggests that Ontario strains are phylogenetically related to strains found in the EWGLI dataset. Phylogenetic cluster I (n = 39) included the epidemic strain ST1 and 7 STs of sporadic cases. With 114 isolates and 46 STs, cluster II was the largest and most diverse group from the dataset. In this cluster, Ontario outbreak strains ST37 and ST211 were subgrouped with ST36 (Philadelphia strain). Cluster III comprised 8 STs and 41 isolates. With the exception of ST222 (n = 13), none of the STs grouped in this cluster were reported to be outbreak strains.

**Figure 2:**
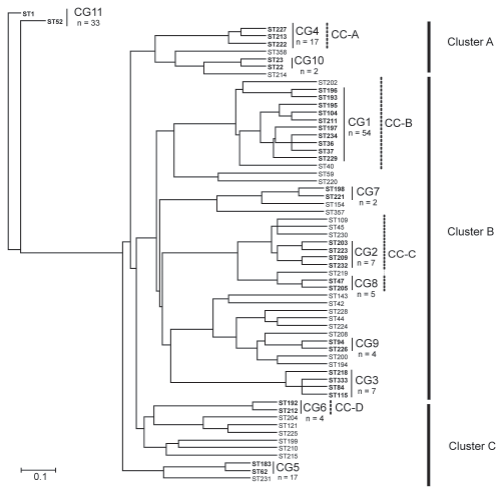
Dendrogram created by the unweighted pair group method with arithmetic mean method based on the 62 allelic profiles of 194 *Legionella pneumophila* serogroup 1 isolates. Clonal groups (CGs) identified by eBURST (http://eBURST.mlst.net) are indicated with solid lines, and STs included in CGs are in **boldface**. Ontario STs included in clonal complexes (CC) identified by comparative eBURST analysis with the European Working Group for *Legionella* Infections database are indicated with dashed lines. The 3 major clusters are indicated on the right of the figure with **bold** lines. The number of strains isolated in Ontario is indicated below CG. Scale bar indicates linkage distances.

Next, the UPGMA algorithm was used to construct a dendrogram based on a matrix of pairwise allelic differences between the 62 STs of our dataset (Figure 2). The topology of the UPGMA dendrogram was partially congruent with the neighbor-joining tree based on allelic sequences. The UPGMA dendrogram contains 3 major clusters of related STs arbitrarily named A, B, and C (Figure 2). Cluster B contains all isolates of cluster II except ST210 and ST199, which grouped with cluster C. In contrast, STs found in clusters I and III were separated into clusters A and C. ST1 and ST52 clustered in a separate branch at the base of the dendrogram, (Figure 2), which suggests that they could be phylogenetically distant from other STs. However, this divergence was not observed with the neighbor-joining method. Based on this finding, for the rest of the analysis, we considered cluster II as a well-defined phylogenetic group and clusters I/III were analyzed as a single group.

### Identification of Clonal Lineages

The eBURST clonal analysis of our strains showed that the province of Ontario presents a semiclonal population with 27 single isolates and 11 clonal groups (CGs) (Figure 2). With 54 isolates and 10 STs, CG1 was the clonal group with the largest number of isolates and STs. This clonal group (27.8% of Ontario isolates) contained STs that were reported elsewhere (ST36, ST37, and ST104) but also STs that were unique to Ontario (ST193, ST195, ST196, ST197, ST211, and ST229). The founder of CG1 was predicted to be ST36 (bootstrap confidence [BC] = 68%), and the predominant single locus variant of this group was ST37 (n = 21). Members of CG1 were recovered from both sporadic and outbreak cases. CG2 (n = 7) only contained isolates with STs that are unique to Ontario and the ancestor of this group was predicted to be ST209 (BC = 28%). All isolates of CG2 were obtained from sporadic cases. Other clonal groups unique to Ontario included CG4 (n = 17), CG6 (n = 4) and CG7 (n = 2). Each of the 5 remaining clonal groups contained only 2 STs with combinations of STs specific to Ontario or previously reported in the EWGLI database.

**Figure 3:**
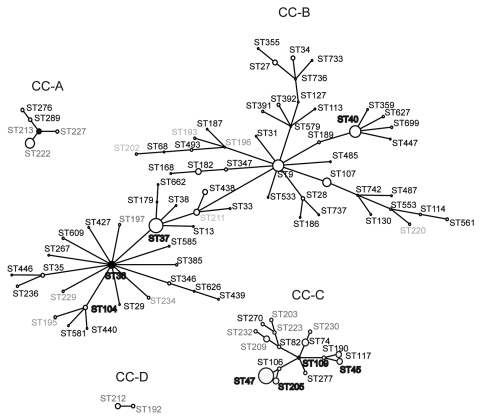
Representation of *Legionella pneumophila* serogroup 1 clonal complexes (CC) A, B, C, D obtained by comparative eBURST (http://eBURST.mlst.net) analysis between the Ontario collection and the European Working Group for *Legionella* Infections database. Each circle represents a single sequence type (ST). Size of the circle is proportional to the number of isolates. Dark circles represent predicted founder of each CC. Labels in **boldface** indicate STs found in both datasets, regular black characters indicate STs absent from the Ontario collection, light gray characters indicate STs exclusively found in Ontario. Solid lines represent single-locus variants.

We next did an eBURST comparative analysis of the SBT dataset from Ontario with the EWGLI database. STs detected in Ontario were only clustered in 17 of the 44 clonal complexes (CC) identified with the EWGLI database. This suggests that >60% of the clonal groups of the EWGLI dataset are absent from Ontario. Comparative eBURST analysis showed that CG1 is part of CC-B, which is the most diversified clonal complex in the international database (59 STs) (Figure 3). In addition to STs included in CG1, Ontario isolates ST40 and ST202 grouped in CC-B. Philadelphia strain ST36 was predicted to be the founder (BC = 91%) of this clonal complex. The high number of STs clustered in CC-B (12 STs) suggests that strains belonging to this group are evolving in Ontario (Figure 2).

Isolates from CG11 grouped within a CC comprising the highest number of EWGLI isolates (n = 490). The predicted founder of this clonal complex is ST1 and despite its high number of isolates, it comprises only 35 STs. Similarly, Ontario CG11 (n = 33) contained only 2 STs which suggests that strains belonging to these clonal groups have limited genetic variability.

Isolates from CG4 (n = 17) grouped with 2 North American clinical strains ST276 and ST289 in CC-A (Figure 3). Another clonal complex of interest was CC-C accounting for 16 STs (Figure 3). This complex included isolates grouped in CG2 and CG8 as well as 7 STs that were not identified in Ontario. Single isolates ST45, ST109, and ST230 from the Ontario dataset also grouped in CC-C. Finally, CC-D of the comparative eBURST analysis was identical to CG6 (Figure 2).

### Temporal Trends of Lp1 Culture-confirmed Legionellosis in Ontario

**Figure 4:**
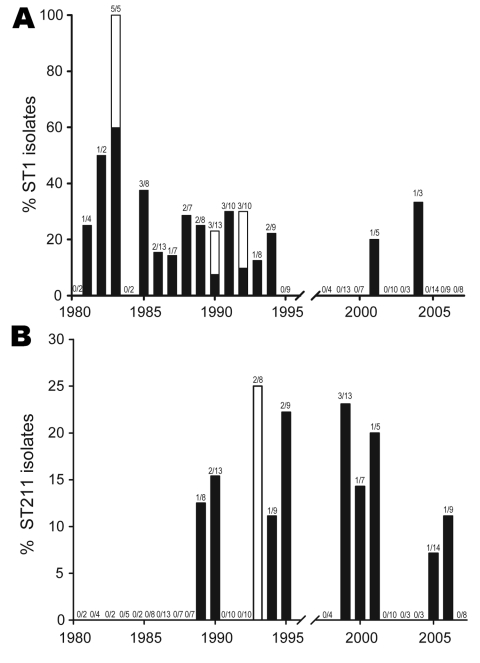
Prevalence of *Legionella pneumophila* serogroup 1 sequence type 1 (ST1) (A) and ST211 (B) endemic strains in Ontario. Black bar sections indicate proportion of strains from isolated cases and white bar sections indicate proportion of isolates from outbreaks.

During 1978–2007, differences could be observed in the distribution of specific STs, clonal complexes and phylogroups. During 1981–1994, ST1 strains were regularly isolated (n = 29) with case numbers ranging from 0 to 5 (peaking in 1983) (Figure 4, panel A). During this time period, ST1 caused 3 outbreaks and a significant increase in ST1 occurrence was observed (incidence rate ratio [IRR] 15.37, 95% confidence interval [CI] 3.67–64.43, p<0.001). In contrast, after 1994, prevalence of ST1 isolates decreased markedly (IRR 0.07, CI 0.02–0.27, p<0.001). On average, from 1978 through 2007, we observed a significant decrease of 9% per year of ST1 strains in Ontario (p<0.001). From 1995 through 2007, only 2 legionellosis ST1 culture-confirmed cases were reported.

In contrast, other STs have emerged in the past 20 years. ST47 was detected 3 times during 2003–2006. This strain was not isolated before 2003 in Ontario and it corresponds to the ST of the Lorraine strain. This emerging strain is highly prevalent in France where it was reported as the cause of 2 major outbreaks (*11*). Two other emerging strains that are unique to Ontario are ST211 and ST222 (Figure 4, panel B). ST211 was first isolated in 1989 accounting for 12.5% of clinical isolates. It was regularly isolated from 1990 through 2006, and sporadic cases peaked in 1999 (23.1%). ST222 was first reported in 1999, and the prevalence of this strain has significantly increased (IRR 1.30 per year, CI 1.12–1.53, p<0.001). Excluding outbreak isolates, ST222 accounted for 11.1% to 15.4% of clinical isolates in 1999, 2000, 2006, and 2007.

**Figure 5:**
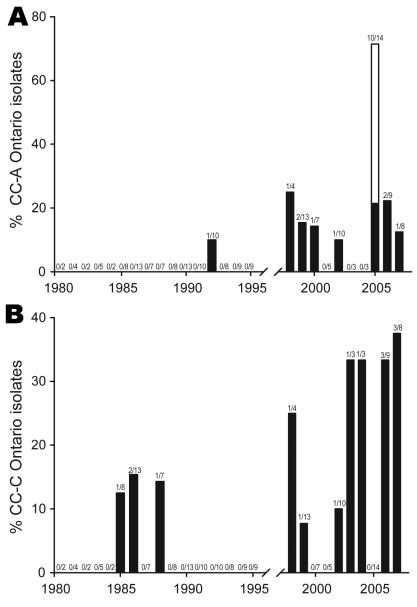
Incidence of Ontario *Legionella pneumophila* serogroup 1 isolates from clonal complexes (CC) A and C. CC-A (A) and CC-C (B) were identified by eBURST (http://eBURST.mlst.net) comparative analysis using the Ontario and the European Working Group for *Legionella* Infections international databases. Black bar sections indicate proportion of strains isolated during sporadic cases. White bar sections indicate proportion of outbreak isolates.

At the clonal complex level, some groups of strains have recently emerged in the province of Ontario. Ontario strains of CC-A were not detected in Ontario before 1992 and oscillated from 10% to 25% from 1998 through 2007 (Figure 5, panel A). This observation is consistent with the emergence of ST222, which is a major contributor of this clonal complex. Chronological evolution of Ontario isolates belonging to CC-C is also noteworthy because it appears to be an emerging clonal complex (Figure 5, panel B). From 1985 through 1988, the maximum incidence of isolates from this clonal complex was 15.4%. CC-C isolates were not reported in 1989 and 1995, but the incidence of CC-C isolates gradually increased from 1998 until 2007 (when it peaked at 37.5%).

### Geographic Distribution of Lp1 Clinical Strains Isolated in Ontario

The geographic distribution of some individual STs, clonal complexes and phylogenetic clusters was not homogenous. With geographic ratios between 37.5% and 100%, the 7 STs that caused >10 clinical cases were all prevalent in the Toronto OHR. ST1 was widely distributed: South West, Central South, Central East, East, and Toronto OHRs. ST37 was found in all OHRs except the East OHR. ST62 was also homogeneously distributed in all OHRs with the exception of the North OHR. In contrast, distributions of ST36 and ST42 were not homogenous because they were only identified in the Central South, Central West, East, and Toronto OHRs. Distribution of ST211 was limited to the Toronto OHR. From 1978 through 2006, ST211 was identified 8 times in the same hospital. Excluding suspected linked cases, ST211 was exclusively detected 7 times in the Toronto OHR. Despite their recent emergence in 1999, ST222 strains were reported in multiple OHRs, including South West, Central West, Toronto, and East.

Comparative eBURST analysis between Ontario and EWGLI datasets grouped Ontario single isolates ST208 (n = 2) with ST257. ST257 was identified in a clinical case of legionellosis from New Hampshire, USA. This small clonal complex may be geographically restricted to eastern North America. Similarly, geographic distribution of CC-A (CG4, ST276, and ST289) was restricted to eastern North America. ST276 and ST289 were only reported in the states of New York and Connecticut (*23*). With the exception of the North OHR, strains belonging to CC-B were reported in all OHRs, although a high prevalence of CC-B isolates were identified in the Toronto OHR (63.2%). Without significant geographic prevalence, CC-C isolates were identified in all OHRs. CC-D comprised STs only reported in Ontario (Figure 3).

**Figure 6:**
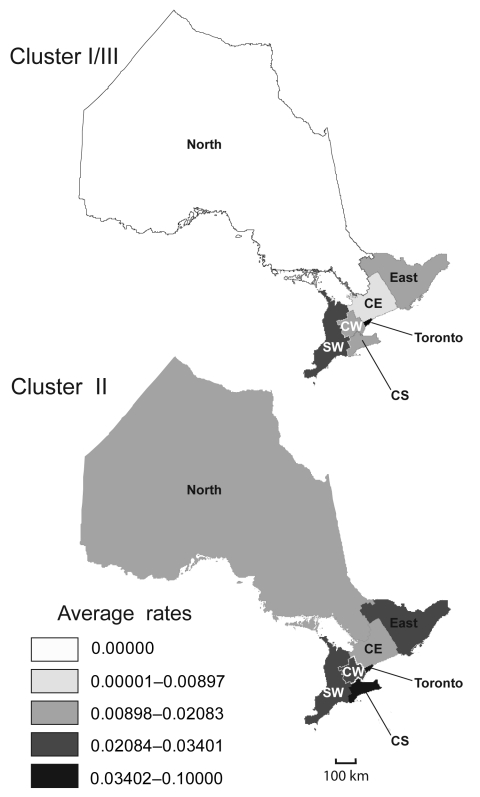
Geographic distribution of phylogenetic clusters II and I/III from 1990 through 2007. Rates are cases of infection with *Legionella pneumophilia* serogroup 1 clones per 100,000 persons per year. The province of Ontario was divided into 7 health regions (OHRs) with populations ranging from ≈0.5 to 2 million persons: Toronto, South West (SW), Central South (CS), Central West (CW), Central East (CE), East, and North.

Geographic distributions of Ontario major phylogenetic groups were analyzed by mapping average rates of culture-confirmed Lp1 cases according to OHRs from 1990 through 2007 (Figure 6). Rates of clusters based on sequence-based types and phylogroups appeared to be partially dependent on geographic location. As expected from our distribution analyses of individual STs and clonal complexes, the Toronto OHR was more likely to have legionellosis cases caused by cluster I/III (0.062/100,000 persons/year) and cluster II (0.08/100,000 persons/year) than all other OHRs. With rates ranging from 0.02/100,000 person years for the North OHR to 0.08/100,000 person-years for the Toronto OHR, isolates from cluster II were unevenly reported in all OHRs. In contrast, legionellosis caused by cluster I/III were not identified in the North OHR and the rate for the Central OHR was only 0.01/100,000 person-years, which is 3.3× less than the rate reported for cluster II.

## Discussion

This report represents the first large-scale population-based SBT analysis of Lp1 clinical isolates within North America over a 30-year period. Sixty-two STs were identified among the isolates of the Ontario collection, which reflects a high degree of genetic diversity of Lp1 clinical isolates. Forty-one STs were unique to Ontario. Thus, the population of clinical Lp1 of this province consists of a combination of widely distributed and local isolates.

Although most sporadic cases were caused by isolates with a unique ST, some STs, like ST1, were found to be responsible for sporadic cases and outbreaks cases. This finding is in agreement with the recent identification of the Paris strain in sporadic and outbreak cases (*24*). Moreover, we have identified additional clones, specifically ST37, ST211, ST222, and ST226, which were also detected in sporadic and outbreaks cases. Although some small clonal groups such as CG2 exclusively comprised sporadic cases, our comparative analysis found no specific correlation between clonal complexes or phylogenetic clusters and ability to cause sporadic or outbreak cases.

A notable finding of our study is the high proportion of ST1 isolates identified, which supports the hypothesis that some specific Lp1 clones have gained widespread dissemination. This hypothesis was proposed after analyses of protein polymorphism and pulsed-field gel electrophoresis showed similar patterns from isolates distributed worldwide (*25*,*26*). Recently, the Paris strain was suggested to be one of these worldwide distributed strains because it has been identified in many European countries in patients and environmental samples (*24*,*27*). In France, the Paris strain was identified in 12.2% of culture-confirmed cases from 1998 through 2002, and it was shown to be the most prevalent endemic strain (*28*). Our study suggests that the notion of worldwide distributed strains could be broadened to include other sequence types such as ST36 or ST37. These 2 STs have been reported in European countries for clinical and environmental isolates and comprised 16% of STs from culture-confirmed cases in Ontario.

In previous studies, the clinical predominance and large distribution of ST1 suggested that it is a stable clone, well adapted to environmental survival or to host infection (*28*,*29*). Surprisingly, although ST1 was identified in 16.5% of the culture-confirmed cases of legionellosis over the past 30 years, our study also shows that the incidence of ST1 strains has decreased dramatically during the past 12 years. Because clonal analysis suggests that ST1 presents a limited genetic variability in our geographic area, we can hypothesize that its ability to colonize the environment or to be isolated by culture or its virulence might have been impaired in the recent years. In contrast, endemic clonal groups and clinical strains like ST211 have emerged in our geographic area in the past 15 years. A surveillance study recently reported a new endemic Lorraine strain (ST47) emerged in France (*11*). ST47 was only recovered 3 times over 30 years in Ontario, but CC-C, comprising ST47, is an emerging clonal complex in Ontario. Our analysis suggests that, globally, ST1 strains are being replaced by other emerging strains or clonal complexes.

Geographic distribution analysis of culture-confirmed population rates suggests that strains from cluster II are largely distributed in Ontario, whereas clusters I/III were mostly reported in the OHRs in close proximity to Lake Ontario. This finding could reflect differences in ecologic niches (either combined with degree of adaption of organisms to cause human disease or not). Some endemic emerging STs and clonal groups are exclusively detected in Ontario, in eastern North America, or in both. In the United States, the census regions with highest incidence rates for legionellosis are East North central and Middle Atlantic, at the proximity of the Great Lakes (*30*). *Legionella* species are abundant in surface waters and the Great Lakes ecosystem might represent an ideal ecologic niche for these bacteria. This hypothesis is in agreement with the identification of clonal complexes comprising isolates exclusively originating from eastern North America. This finding contrasts with findings of a recent population structure analysis of *L. pneumophila* that used allelic profiles from the EWGLI database that could not identify eBURST groups containing profiles originating from a single geographic area (*22*).

In conclusion, we showed that the population of clinical Lp1 in the province of Ontario is a combination of worldwide distributed and local strains. Our population of isolates might represent more severe cases as human respiratory samples are more frequently taken from patients requiring hospitalization, but the decreased prevalence of some clones and the emergence of local group of isolates suggest that the population of Lp1 has evolved or adapted to its environment during the past 30 years. Further research is required to explain the changing incidence of these STs and to investigate the fitness of emerging strains or clonal groups. Outcomes of this research will be helpful to improve surveillance programs for legionellosis as well as to ensure adequacy of clinical testing procedures with circulating strains.

## Appendix

**Table A1:** Sequence types and allelic profiles of clinical *Legionella pneumophila* serogoup 1 isolates from Ontario, 1978–2007

Sequence type	Allelic profile	Isolation dates	No. isolates	% Outbreak isolates	Unique to Ontario?
1	1,4,3,1,1,1,1	1981–2004	31	19.35	No
22	2,3,6,10,2,1,6	1987	1	–	No
23	2,3,9,10,2,1,6	1993	1	–	No
36	3,4,1,1,14,9,1	1986–2005	10	–	No
37	3,4,1,1,14,9,11	1985–2007	21	9.52	No
40	3,6,1,14,14,9,11	1991–1992	2	–	No
42	4,7,11,3,11,12,9	1989–2007	10	–	No
44	4,8,11,10,10,12,2	1984	1	–	No
45	5,1,22,26,6,10,12	2004	1	–	No
47	5,10,22,15,6,2,6	2003–2006	3	–	No
52	1,10,3,1,1,1,1	1991	1	–	No
59	7,6,17,3,13,11,11	1982–1992	4	–	No
62	8,10,3,15,18,1,6	1986–2006	16	–	No
84	12,9,2,5,3,17,15	1980	1	–	No
94	12,8,11,5,20,12,2	1999	1	–	No
104	3,10,1,1,14,9,1	1995	1	–	No
109	5,1,22,15,6,10,6	2007	1	–	No
115	12,29,2,.5,3,17,15	2006	1	–	No
121	2,10,3,10,9,4,6	2005	1	–	No
143	4,17,11,23,5,12,19	1989	1	–	No
154	11,14,16,16,15,13,2	2000	1	–	No
183	8,10,9,15,18,1,6	1980	1	–	Yes
192	2,10,5,5,35,5,6	1981	1	–	Yes
193	3,10,1,28,14,9,6	1981	1	–	Yes
194	12,8,11,2,11,12,2	1981	1	–	Yes
195	3,10,3,1,14,9,1	1984–1992	2	–	Yes
196	3,10,1,28,14,9,11	1985	1	–	Yes
197	3,4,1,1,29,9,1	1985	1	–	Yes
198	11,14,3,1,15,13,9	1986	1	–	Yes
199	6,10,17,28,4,14,11	1986	1	–	Yes
200	12,15,11,10,11,12,2	1986	1	–	Yes
202	3,13,1,28,1,9,3	1987	1	–	Yes
203	5,8,22,10,6,10,2	1988	1	–	Yes
204	1,10,3,3,9,4,9	1988	1	–	Yes
205	5,10,22,15,6,1,6	1985–1986	2	–	Yes
208	12,8,11,5,8,12,22	1986–1990	2	–	Yes
209	5,1,22,10,6,10,19	1986–2007	4	–	Yes
210	6,10,15,10,9,14,10	1988–2003	2	–	Yes
211	3,10,1,1,14,9,11	1989–2006	15	13.33	Yes
212	2,10,5,5,35,5,9	1991–2007	3	–	Yes
213	2,19,5,10,18,1,2	1992–2005	3	–	Yes
214	2,10,9,10,10,1,6	1992	1	–	Yes
215	2,10,14,10,21,14,3	1993	1	–	Yes
218	12,9,2,21,3,17,15	1993	1	–	Yes
219	5,10,22,10,6,5,6	1994	1	–	Yes
220	3,6,1,3,9,11,9	1995	1	–	Yes
221	11,14,16,1,15,13,9	1995	1	–	Yes
222	2,19,5,10,18,1,10	1999–2007	13	53.85	Yes
223	5,1,22,10,6,10,2	1998	1	–	yes
224	4,8,11,16,42,12,2	1999	1	–	Yes
225	2,10,3,25,9,4,13	2001	1	–	Yes
226	12,8,11,26,20,12,2	2001–2002	3	66.66	Yes
227	2,19,3,10,18,1,2	2002	1	–	Yes
228	4,8,22,16,29,12,2	1995–2002	2	–	Yes
229	5,4,1,1,14,9,1	2002	1	–	Yes
230	5,1,22,30,6,10,10	2002	1	–	Yes
231	8,10,3,15,21,12,20	2007	1	–	Yes
232	5,1,22,23,6,10,19	2007	1	–	Yes
234	3,4,1,1,14,9,9	1999	1	–	Yes
333	12,31,2,5,3,17,15	1988–1995	4	–	Yes
357	11,14,32,3,7,33,9	2002	1	–	Yes
358	12,3,9,13,52,1,6	1998	1	–	Yes
